# ﻿Resolving the *Drymoniakillipii* (Gesneriaceae) complex results in a new species from the northwestern Andes of South America

**DOI:** 10.3897/phytokeys.248.123248

**Published:** 2024-10-31

**Authors:** Laura Clavijo, John L. Clark

**Affiliations:** 1 Universidad Nacional de Colombia, Sede Bogotá, Facultad de Ciencias, Instituto de Ciencias Naturales, Bogotá, D.C., 111321, Colombia Universidad Nacional de Colombia Bogotá Colombia; 2 Marie Selby Botanical Gardens, Sarasota, FL, USA Marie Selby Botanical Gardens Sarasota United States of America

**Keywords:** Andes, Chocó biogeographic region, Colombia, Columneinae, Ecuador

## Abstract

A new species, *Drymoniaquadrangulata* Clavijo & J.L.Clark, **sp. nov.** (Gesneriaceae, Columneinae), is described from the western Andean slopes of southern Colombia and northern Ecuador. The new species has been historically confused with *D.killipii*, an endemic species to Colombia, restricted to the lowlands of the Chocó biogeographic region in the departments of Cauca, Chocó, and Valle del Cauca. These two species have large foliaceous calyx lobes that cover at least half of the corolla tube, and tubular-infundibuliform corollas. The new species differs by strigose quadrangulate and sometimes winged stems, leaves reticulate abaxially with obtuse to subcordate bases, midveins green, corolla lobes white to yellow with margins incised to short laciniate, and indehiscent berry fruits. Digital photographs, detailed morphological comparisons with the similar species, and an IUCN preliminary risk extinction assessment are provided for the new species.

## Abstract (es)

Se describe una nueva especie, *Drymoniaquadrangulata* Clavijo & J.L.Clark, **sp. nov**. (Gesneriaceae, Columneinae), de la vertiente occidental de los Andes del sur de Colombia y el norte de Ecuador. La nueva especie ha sido históricamente confundida con *D.killipii*, una especie endémica de Colombia, restringida a las tierras bajas del Chocó biogeográfico en los departamentos de Cauca, Chocó y Valle del Cauca. Estas dos especies tienen un cáliz foliáceo que cubre al menos la mitad del tubo de la corola y corolas tubulares-infundibuliformes. La nueva especie se distingue por sus tallos estrigosos, cuadrangulares y a veces alados, hojas reticuladas en el envés con bases obtusas a subcordadas, nervadura central verde, lóbulos de la corola blancos a amarillos con márgenes incisos a cortamente laciniados y frutos en bayas indehiscentes. Se proporcionan para la especie nueva fotografías digitales, comparaciones morfológicas detalladas con las especies similares y una evaluación preliminar de riesgo de extinción de acuerdo con los criterios de la UICN.

## ﻿Introduction

*Drymonia* Mart. ranks as the third-largest genus within neotropical Gesneriaceae, surpassed only by *Columnea*, with 210+ species, and *Besleria*, with 175+ species (Clark et al. 2020; GRC 2024). Based on our ongoing revision of the genus, *Drymonia* comprises 87 described species, most of them concentrated in the northern Andes and the Chocó biogeographic region, especially in Colombia (40 species) and Ecuador (38 species). Molecular sequence data strongly supports the monophyly of *Drymonia* (Clark et al. 2015; Ogutcen et al. 2021). *Drymonia* is classified in the subtribe Columneinae that represents 16% (approximately 525+ spp.) of the total species diversity in the Gesneriaceae (Weber et al. 2013, 2020).

*Drymonia* is a heterogenous genus characterized by a diverse array of leaf shapes, indumenta, corolla shapes and colors, and fruit types. Species within the genus range from herbs, sub-shrubs, shrubs, vines or lianas. The scandent habit is best summarized as a nomadic climber (Moffett 2000) characterized as germinating on the ground, ascending onto other plants using scandent stems or adventitious roots, and potentially shedding older stem parts in the process of ascent (Zotz 2013). Other common habits in *Drymonia* include epiphytes (facultative or obligate) or terrestrial. Corollas vary from campanulate to tubular, infundibuliform or hypocyrtoid (i.e., constricted apically with a ventral pouch) with a broad diversity of limb colors and margins. The corolla lobe margins range from entire or crenate, to laciniate or fimbriate. Anthers typically dehisce by basal pores, historically a defining character for *Drymonia* (Wiehler 1983); however, at least two clades within the genus exhibit longitudinal dehiscence, indicating that poricidal dehiscence has been likely lost twice in *Drymonia* (Clark et al. 2006, 2015). Fruit shapes in *Drymonia* are classified into the following four types (Clark and Clavijo 2022): 1) fleshy display capsules (no separate endocarp), 2) fleshy capsules with tardily dehiscent endocarps, 3) fleshy capsules with non-dehiscent endocarps, and 4) berries.

**Figure 1.**
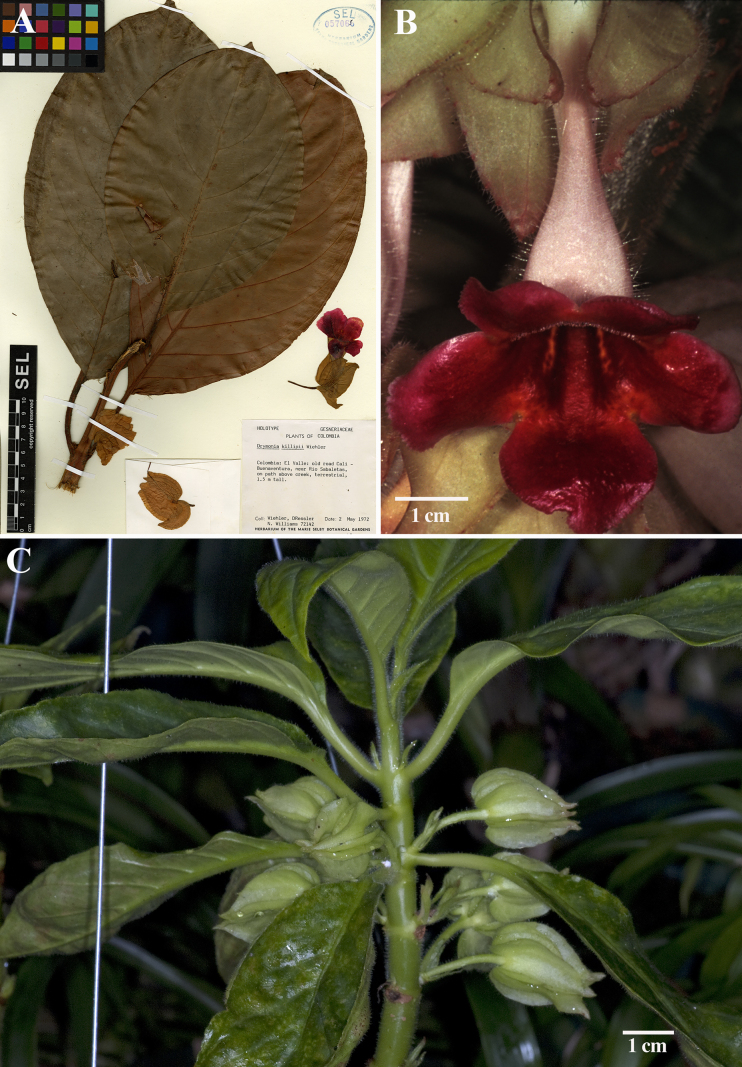
*Drymoniakillipii* Wiehler **A** holotype at SEL (*H. Wiehler 72142*) **B** dorsal view with front of corolla **C** cultivated collection from the Atlanta Botanical Garden (leaves are smaller due to cultivation conditions) (**B** from *H. Wiehler* live collection W-1726 **C** from *J.L. Clark 10048*).

Our ongoing studies on the genus *Drymonia* based on extensive fieldwork and revision of herbarium collections have allowed us to identify a significant number of collections misidentified as *Drymoniakillipii* Wiehler (Wiehler 1977) (Fig. 1). Subsequently, our observations of species concepts based on herbarium research were confirmed with our preliminary DNA sequence data from two nuclear and two plastid regions, supporting *D.quadrangulata* as not closely related to *D.killipii* (Clavijo 2016) and, therefore, recognized here as a new species. Hans Wiehler collected *Drymoniakillipii* in the Colombian department of Valle del Cauca in 1972 (*H. Wiehler 72142*), brought it into cultivation, and later described it as a new species (Wiehler 1977). Living plants of *Drymoniakillipii* were cultivated at Marie Selby Botanical Gardens in Sarasota (Florida, USA) until they were lost around 2019. Fortunately, Selby Gardens shared cuttings with Atlanta Botanical Garden where individuals of this species are currently thriving (David Ruland pers. comm.). *Drymoniakillipii* is a distinctive species, featuring terete stems covered by a hirsute indumentum that becomes glabrescent with age, and large leaves (up to 45 cm long) with suppressed inter-secondary and tertiary venation abaxially (Fig. 1). It also has large foliaceous calyces and a tubular-infundibuliform corolla with a royal purple to maroon limb suffused with lemon-yellow toward the throat (Fig. 1B). Notably, this species is characterized by fragrant flowers described as having a lemon scent (Wiehler 1977). It is a narrow endemic, found in the lowlands of the Chocó biogeographic region of Cauca, Chocó, and Valle del Cauca (Colombia) and is only known from 29 collections, most of which were made in the past century.

Here, we describe a new species that has been confused with *Drymoniakillipii*, clarify their morphological differences, and compare the new species with similar congeners (Table 1). The description of *D.quadrangulata* is based on the study of herbarium specimens, living plants in their natural habitats, and photographic images; the terminology used in the description follows Beentje (2016).

**Table 1.** General geographic distribution and comparison of morphological characters to differentiate *Drymoniaquadrangulata*, *D.killipii*, *D.chiribogana*, and *D.lanceolata*.

	*D.quadrangulata*	*D.killipii*	*D.chiribogana*	*D.lanceolata*
**Habit**	Terrestrial, rarely a nomadic climber	Epiphytic, rarely terrestrial	Epiphytic	Terrestrial
**Stems**	Branched, rarely unbranched	Branched	Sparsely branched	Unbranched, rarely branched
Indument	Strigose, glabrescent with age	Hirsute, glabrescent with age	Glabrescent	Strigose, glabrescent with age
Surface	Smooth	Smooth	Papyraceous	Papyraceous
Shape	Quadrangular to strongly angulate, sometimes winged	Terete	Terete to subquadrangular	Quadrangular to angulate
**Leaf pairs**	Equal to subequal	Subequal to unequal	Subequal to unequal	Equal
**Petiole length**	(2.2–)5.2–15.0 cm	2.0–6.4 cm	1.8–10 cm	2.5–6(–14.4) cm
Indument	Strigose	Hirsute	Glabrescent	Strigulose
**Blade shape**	Elliptic to ovate	Elliptic to obovate	Oblanceolate-elliptic, asymmetrical	Elliptic
Base	Obtuse, rounded or subcordate	Cuneate	Cuneate, oblique	Cuneate to attenuate
Margin	Serrulate to serrate	Subentire	Entire to subentire	Serrate
Indument abaxially	Glabrescent	Hirsute to glabrescent	Glandular trichomes sunken into the epidermis	Puberulous to strigulose
Veins abaxially	Reticulate	Suppressed	Suppressed	Reticulate
Veins indument	Minutely puberulent	Hirtellous to hirsute	Glabrescent	Strigose to tomentose
**Pedicel indument**	Hirsute with glandular trichomes	Glabrescent	Strigose to strigulose	Strigulose
**Calyx lobes color**	Green, green with reddish or maroon toward margins	Yellow green	Light green, often suffused with purple or maroon	Green
Fusion	Nearly free	Nearly free	Nearly free	Free
Shape	Lanceolate	Lanceolate	Ovate	Lanceolate to ovate
Apex	Attenuate	Acute to attenuate	Acute	Long acuminate
Base	Cordate	Cordate	Strongly cordate	Cordate
Margin	Entire to minutely serrulate, folding outwards	Subentire or ciliate, folding outwards	Subentire, folding outwards	Entire, flat
Indument abaxially	Glabrescent, strigulose at base	Sparsely hirsute with scattered glandular trichomes	Glabrescent	Puberulous to strigose
**Corolla length**	4.3–6.5 cm	5.5–7.5 cm	3.2–4.3 cm	(3.6–)4.5–5.1 cm
Tube indument	Outside puberulous, inside glabrescent	Outside sparsely pilose, inside glabrous	Glabrous	Outside puberulous, inside glabrescent
Throat color	Yellow	Lemon-yellow, with brown spots and dots	Yellow with red lines to red	Yellow
Limb color	White to yellow	Royal purple to maroon	Magenta	White, suffused with light yellow at base
Corolla lobes margin	Incised to short laciniate	Subentire	Subentire to incised	Long-fimbriate
Ovary indument	Puberulous to velutinous	Hirsute with glandular trichomes	Glabrous	Glabrous to strigulose
Style indument	Puberulous to velutinous	Hirsute with glandular trichomes	Glabrous	Strigillose
Stigma shape	Stomatomorphic	Stomatomorphic	Stomatomorphic	Deeply bilobed
**Fruit**	Berry	Bivalved fleshy capsule	Bivalved fleshy capsule	Berry
**Distribution**	Northwestern Ecuador and Southwestern Colombia	Endemic to Colombia (Chocó and Valle del Cauca)	Endemic to Ecuador	Widely distributed from Costa Rica to Ecuador
**Elevation**	250–2250 m	0–200 m	80–1800 m	140–2400 m

## ﻿Taxonomic treatment

### ﻿ Drymonia quadrangulata

Taxon classificationPlantaeLamialesGesneriaceae

Clavijo & J.L.Clark sp. nov.

D819F3D4-A661-5A5D-9098-F230DE4E9C16

urn:lsid:ipni.org:names:77351102-1

Figs 2 , 3

#### Diagnosis.

Differs from *Drymoniakillipii* by quadrangular to strongly angulate stems in cross-sections vs. terete; stems strigose apically vs. hirsute; blades 12.0–26.7 cm long vs. 20.5–45.0 cm long; corolla lobes white to yellow vs. royal purple to maroon; and fruits indehiscent globose berries vs. bivalved fleshy capsules.

#### Type.

Ecuador. Imbabura: Ibarra. Parroquia: Lita. Unpaved road heading south, near Rocafuerte (between San Gerónimo and La Carolina), accessed via the Lita−San Lorenzo highway (KM 98), road recently developed through the SolGold mining concession (Alpala); 0.7526389°N, 78.373889°W; 1550–1650 m; 23 Jul 2022; *J.L. Clark, Álvaro Pérez, Francisco Tobar & Russell Clark 17079* (holotype: QCA; isotypes: COL, CUVC, E, F, G, MO, NY, QCNE, SEL! [barcode: SEL086219], US).

#### Description.

Terrestrial herb or shrub, 0.6–1.5 m tall. ***Stem*** scandent basally and then erect, usually branched, adventitious roots usually absent, quadrangular in cross-section to strongly angulate, sometimes winged, 4.2–10.5 mm in diameter, herbaceous to succulent, green to green with maroon spots, smooth, strigose apically, becoming glabrescent with age, lenticels sometimes present, internodes 2.2–10.2 cm long, reduced toward the apex. ***Leaves*** opposite, decussate, equal to subequal in a pair; petiole (2.2–)5.2–15.0 cm long, green with maroon spots, terete in cross-section, grooved, flattened at base, pairs of petiole bases fused together forming a perfoliate-like flap or wing, 1–2 mm wide, petiole enations present at base of petiole, strigose in apical leaves, glabrescent in basal leaves; blade elliptic to ovate, 12.0–26.7 × 5.2–18.5 cm, coriaceous, green adaxially, light green suffused with maroon abaxially, brown-maroon when dried, apex acute to acuminate, base obtuse, rounded or subcordate, margin serrulate to serrate, minutely strigose to glabrescent adaxially, glabrescent abaxially, 5–7 pairs of main lateral veins, minutely puberulent, reticulated, evident on both surfaces but more so abaxially. ***Inflorescence*** axillary, a reduced pair-flowered cyme with 1–6 flowers per inflorescence; bracts usually caducous, 7.0–16.9 × 2.0–3.7 mm, light green suffused with maroon to mostly maroon, lanceolate to oblong, apex acute, base obtuse, margin entire, glabrescent adaxially, strigulose abaxially; peduncle absent. ***Flowers*** non-resupinate; pedicel erect to perpendicular 9.5–42.0 mm long, green, strigulose, enations scattered along the pedicel. Calyx green, green with reddish or maroon margins, or mostly maroon, membranaceous, persistent in fruit, calyx lobes 5, 4 nearly equal, nearly free, fused at the base for 0.7–2.0 mm, with margins overlapping at least half their length, lanceolate, apex attenuate, base cordate, margins entire to minutely serrulate, glabrescent adaxially, glabrescent but strigulose at base abaxially, ventral and lateral lobes 25.4–53.2 × 10.3–36.0 mm, dorsal lobe smaller, 25.0–37.6 × 8.4–19.0 mm. Corolla zygomorphic, protandrous, oblique to perpendicular relative to calyx, tubular, 42.7–64.6 mm long; tube gibbous at base, slightly constricted above base and wider at the middle, 30.8–49.5 mm long, 5.8–9.9 mm wide at constriction above base, 10.7–16.6 mm wide at the middle, outside white and puberulous, inside light yellow and glabrescent, with glandular trichomes toward throat on dorsal surface, nectary chamber 4.7–10.2 mm long; throat 9.1–15.1 mm in diameter, outside white and puberulous, inside yellow, ventrally darker and thickened forming a groove, dorsally with glandular trichomes; corolla lobes 5, subequal, white to yellow, orbicular, apex rounded, margin incised to short laciniate, glabrous adaxially, glabrous to strigulose abaxially, ventral lobe larger, straight to spreading, 14.2–23.9 × 12.2–33.4 mm, lateral lobes spreading, 12.1–23.1 × 12.7–25.0 mm, upper lobes, 10.3–22.3 × 10.3–21.6 mm. Androecium of 4 didynamous stamens, included, filaments 29.0–35.0 mm long, adnate to the corolla tube for 5.1–14.0 mm, white, glabrous, coiling after anthesis, staminode absent; anthers oblong, sagittate, coherent by the lateral walls, dehiscence by basal pores that develop into longitudinal slits, 4.2–7.0 × 0.7–2.0 mm. Gynoecium with a single dorsal nectary gland, oval, apex irregularly acute to obtuse, 1.7–2.7 mm long, white, glabrous; ovary superior, 4.2–10.0 × 2.1–6.7 mm, ovate, yellow to yellow-green, puberulous to velutinous; style included, 23.5–35.1 mm long, puberulous to velutinous, reddish at base, white apically; stigma stomatomorphic, white. ***Fruit*** a berry, 6.8–17.0 × 5.5–16.5 mm, globose, yellow at maturity. ***Seeds*** numerous, 0.8–1.0 × 0.4–0.5 mm, brown, fusiform, longitudinally ridged.

#### Phenology.

Collected with flowers throughout the year. Fruits collected in May and June.

#### Etymology.

**Figure 2.**
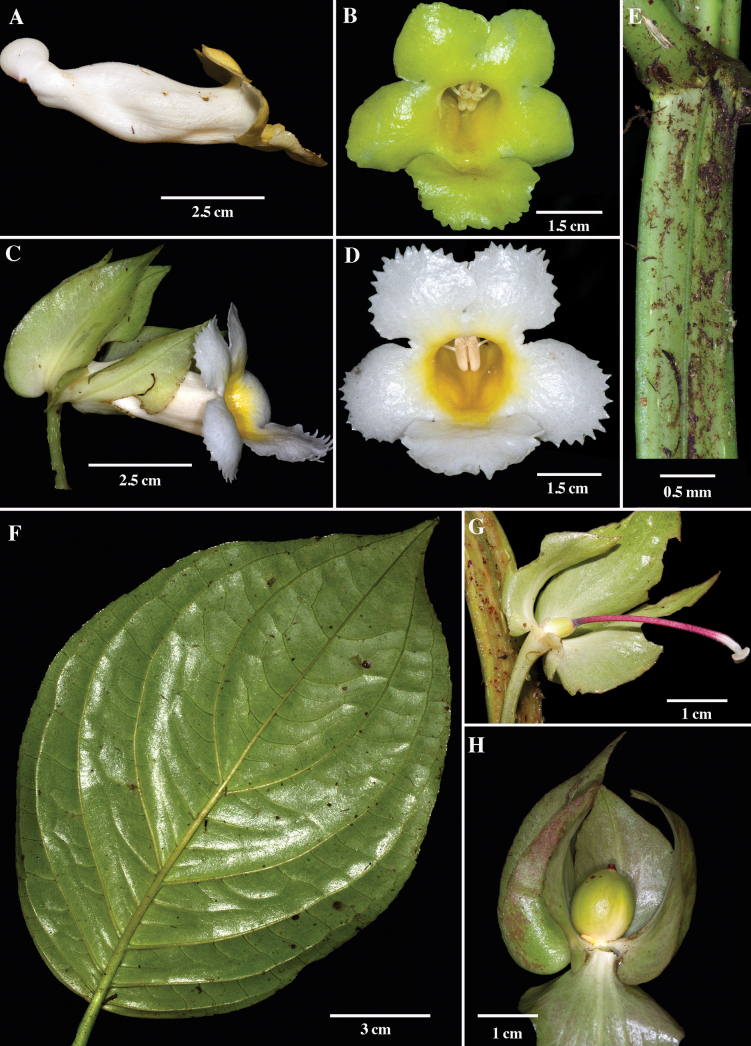
*Drymoniaquadrangulata* Clavijo & J.L.Clark **A** lateral view of corolla **B** front view of flower (yellow form) **C** lateral view of flower **D** front view of flower (white form) **E** quadrangular stem **F** abaxial surface of leaf **G** gynoecium **H** lateral view of immature fruit (**A, G** from *L. Clavijo et al. 1879***B** from *J.L. Clark 13609***C, E***J.L. Clark et al. 10344***D***J.L. Clark 17079***F** from *J.L. Clark 18123***H** from *J.L. Clark 16320*). Photos: **A, G** by L. Clavijo, **B–F, H** by J.L. Clark.

**Figure 3.**
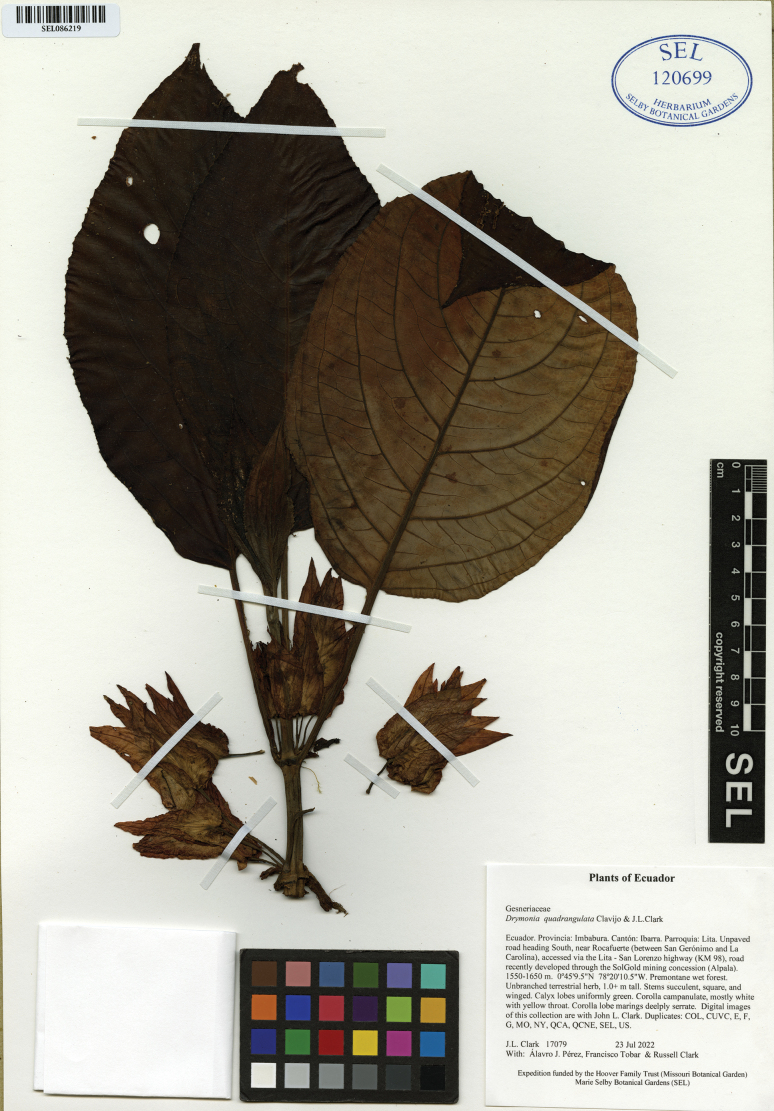
Isotype of *Drymoniaquadrangulata* Clavijo & J.L.Clark (SEL).

The specific epithet is in reference to the quadrangular to strongly angulate stem cross-section which is occasionally winged (Figs 2E, 3).

#### Distribution and preliminary conservation assessment.

**Figure 4.**
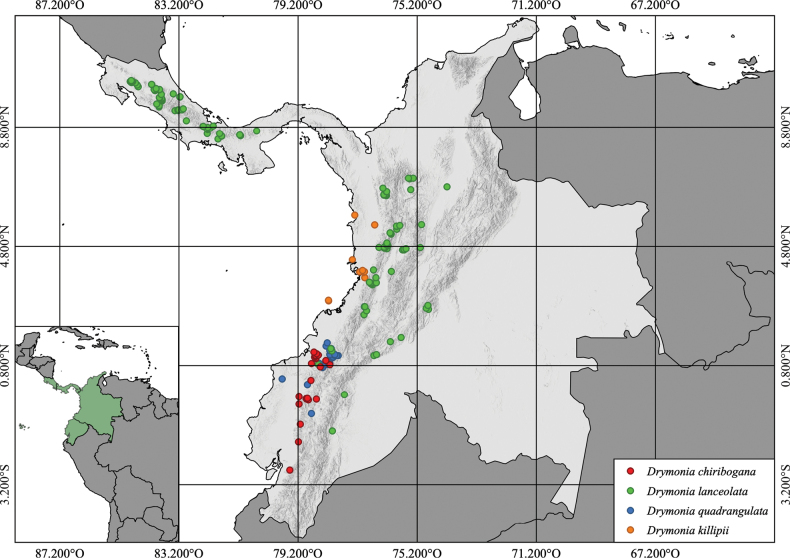
Distribution map of *Drymoniaquadrangulata* (blue circles), *D.killipii* (orange circles), *D.chiribogana* (red circles), and *D.lanceolata* (green circles).

*Drymoniaquadrangulata* has been recorded from the western slopes of the Andes in southwestern Colombia (Cauca and Nariño) and northern Ecuador (Carchi, Esmeraldas, Imbabura, and Pichincha), between 250 and 2300 m (Fig. 4). It grows in the premontane rainforest, in the transition between the Tropical Andes and the Tumbes-Chocó-Magdalena Hotspots (Mittermeier et al. 2004), in a region characterized by high precipitation, which in some areas may reach more than 7000 mm per year (FELCA 2024). In Colombia, this species has been documented in protected areas such as the National Natural Park Munchique, and the Natural Reserves Río Ñambí and La Planada, with few collections in their surroundings. In Ecuador, it has also been found in protected areas such as the Mache-Chindul Ecological Reserve, the Dracula Natural Reserve (Fundación Ecominga), and the Bosque Protector Mashpi (Mashpi Lodge). According to GeoCAT (Bachman et al. 2011), the following values were calculated: EOO = 34,836 km^2^ and the AOO = 132 km^2^. Based on the IUCN Red List Categories and Criteria (2012) and updated criteria in the IUCN Standards and Petitions Committee (2024), the AOO satisfies criterion B2 for Endangered (<500 km^2^), but the other criteria do not support a threatened category. Given the large EOO and the presence of several populations in protected areas, *Drymoniaquadrangulata* is preliminarily assessed as being of Least Concern (LC).

#### Comments.

**Figure 5.**
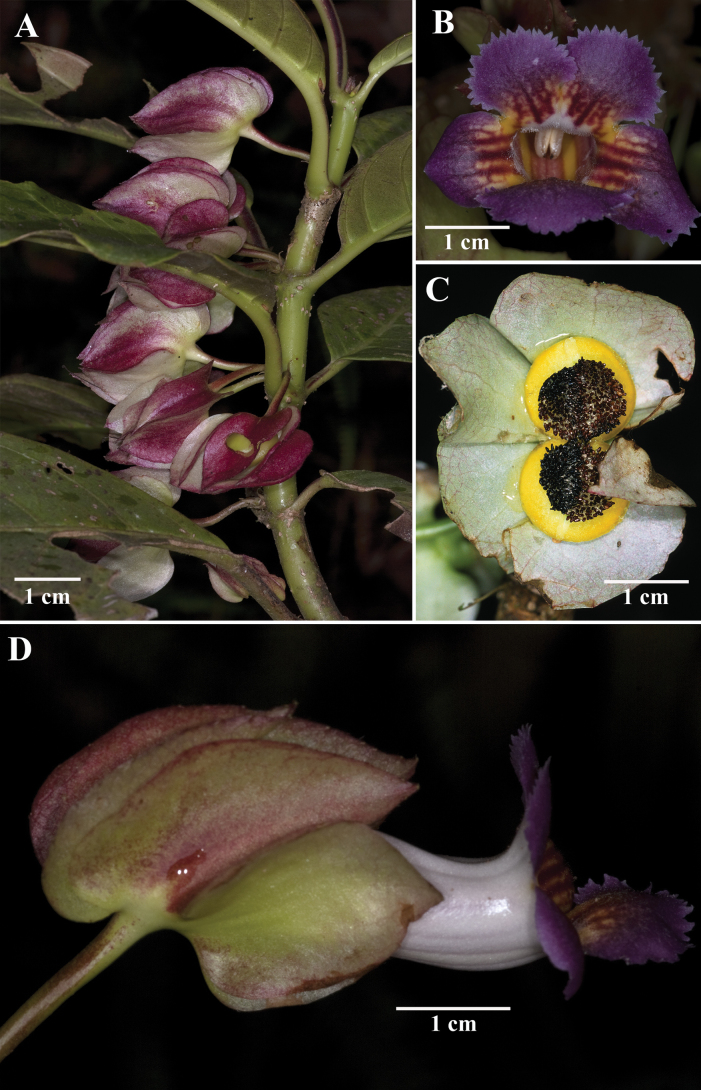
*Drymoniachiribogana* Wiehler **A** habit **B** front view of corolla **C** mature fleshy bivalved capsule **D** lateral view of flower (**A, B, D** from *J.L. Clark et al. 10935***C** from *J.L. Clark et al. 7358*). Photos by J.L. Clark.

**Figure 6.**
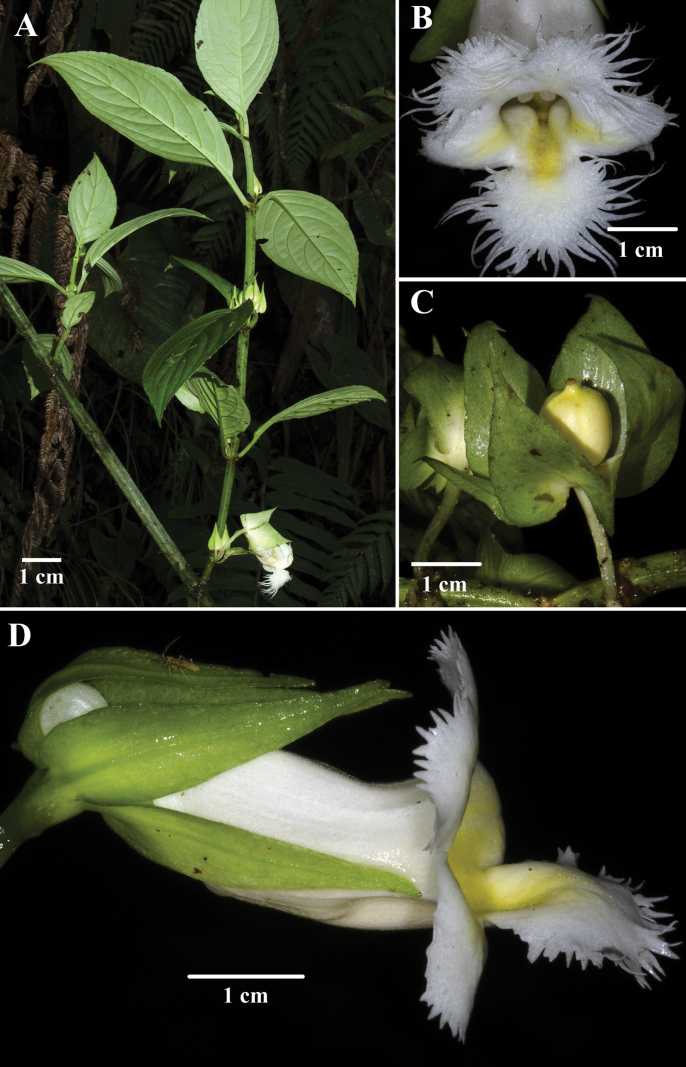
*Drymonialanceolata* (Hanst.) C.V.Morton. **A** erect habit **B** front view of corolla **C** immature fruit **D** lateral view of flower (**A** from *J.L. Clark et al. 13257***B** from *J.L. Clark et al. 13325***C** from *J.L. Clark 13341***D** from *J.L. Clark et al. 13618*). Photos by J.L. Clark.

*Drymoniaquadrangulata* is morphologically similar to *D.killipii* (Fig. 1), *D.chiribogana* (Fig. 5), and *D.lanceolata* (Fig. 6). These taxa possess foliaceous calyx lobes, nearly free, covering at least half of the corolla tube and sometimes the entire tube. The calyx lobe apices are acute to attenuate, with margins folded longitudinally outwards, and tubular-infundibuliform corollas. *Drymoniaquadrangulata* is often collected, but misidentified in most herbaria as *D.killipii*, a rarely collected narrow endemic in Colombia. However, *D.quadrangulata* can be distinguished from *D.killipii* by its strigose indumentum in vegetative and reproductive structures that becomes glabrescent with age (Fig. 2E) vs. hirsute, becoming glabrescent with age (Fig. 1C), stem quadrangular to strongly angulate (Figs 2E, 3), sometimes winged vs. terete (Fig. 1C), petiole of (2.2–)5.2–15.0 cm long vs. 2.0–6.4 cm long, blade base obtuse to subcordate (Figs 2F, 3) vs. cuneate (Fig. 1A), inter-secondary and tertiary venation reticulated (Fig. 2F) vs. obscure or suppressed (Fig. 1A), pedicel and calyx lacking glandular trichomes vs. presence of glandular trichomes, calyx lobes lanceolate (Fig. 2C) vs. ovate (Fig. 1A), corolla lobes white to yellow (Fig. 2B–D) vs. royal purple to maroon, suffused with lemon-yellow toward the throat (Fig. 1B), corolla lobes margin incised to short laciniate (Fig. 2A–D) vs. subentire (Fig. 1B), and indehiscent berry fruits (Fig. 2H) vs. bivalved fleshy capsules. Additionally, Wiehler (1977) noted the fragrant lemon scent of *D.killipii*, while no scent has been recorded for *D.quadrangulata*.

*Drymoniaquadrangulata* can be distinguished from *D.chiribogana* (Fig. 5) by its terrestrial habit vs. epiphytic, stem quadrangular to strongly angulate (Fig. 2E) vs. terete to subquadrangular (Fig. 5A), stem surface smooth when dried vs. papyraceous, blade green with midvein green adaxially vs. green with veins whitish or silvery, blade glabrescent abaxially vs. with glandular trichomes sunken into the epidermis, inter-secondary and tertiary venation reticulated (Figs 2F, 3) vs. obscure or suppressed, calyx lobes lanceolate (Fig. 2C) vs. broadly ovate (Fig. 5D), corolla of 4.3–6.5 cm long (Fig. 2A–D) vs. 3.2–4.3 cm long (Fig. 5D), corolla lobes white to yellow (Fig. 2B–D) vs. magenta, with red or yellow toward the throat (Fig. 5B), corolla lobes margin incised to short laciniate (Fig. 2A–D) vs. subentire to incised (Fig. 5B, D), and berry fruit (Fig. 2H) vs. bivalved fleshy capsule (Fig. 5C).

While *Drymoniaquadrangulata* shares a terrestrial habit, quadrangulate to angulate stems, and berry fruits with *D.lanceolata* (Fig. 6), it can be differentiated by the stem surface smooth when dried vs. papyraceous, blade base obtuse to subcordate (Fig. 2F) vs. cuneate to attenuate (Fig. 6A), veins minutely puberulent abaxially vs. strigose to tomentose, calyx lobes nearly free with margins folded longitudinally outwards vs. free with margins flat (Fig. 6D), corolla lobes incised to short laciniate (Fig. 2A–D) vs. long-fimbriate (Fig. 6B, D), and stomatomorphic stigma vs. deeply bilobed. The morphological differences between *Drymoniaquadrangulata*, *D.killipii*, *D.chiribogana*, and *D.lanceolata* are summarized in Table 1.

#### Specimens examined.

**Colombia – Cauca.** • El Tambo; PNN Munchique, camino a López de Micay, entre El Boquerón y La Cueva; Bosque Pluvial premontano; 2.76816667°N, 76.974361°W; 900–1050 m; 14 Jun 2017; fl; *L. Clavijo, et al. 1868* (COL, CAUP, CUVC) • ibid.; 15 Jun 2017; fl, fr; *L. Clavijo et al. 1879* (COL) • ibid.; PNN Munchique, camino a López de Micay, alrededores de la quebrada Aguaclara, Bosque Pluvial premontano; 2.76816667°N, 76.967417°W; 1045–1220 m; 17 Jun 2017; fr; *L. Clavijo et al. 1900* (COL); • ibid.; bud; *L. Clavijo et al. 1901* (COL). – **Nariño.** • Barbacoas; Corregimiento Altaquer; vereda El Barro, Reserva Natural Río Ñambí; 1.299103°N, 78.084362°W; 1325 m; 4 Dec 1993; *J. Betancur 4518* (COL) • ibid.; 1.28333333°N, 78.066667°W; 1200–1400 m; 19 Apr 2004; *N.R. Salinas 516* (COL) • ibid.; Reserva Natural Río Ñambí, sendero hacía Puente Piedra y bosque al otro lado del río Peje; 1.4523911°N, 78.259865°W; 1400 m; 25 Jul 2011; fr; *L. Clavijo, M. Flores & A. Vasquez 1667* (COL) • ibid.; Reserva Natural Río Ñambí, vertiente occidental andina, bosque fluvial premontano, bosque primario poco intervenido, margen derecha del Río Ñambí; 1.3°N, 78.133333°W; 1325 m; 8 Dec 1993; *J. Betancur 4738* (COL) • ibid.; Reserva Natural Río Ñambí; 1.16666667°N, 78.133333°W; 1325 m; 6 Dec 1993; *P. Franco 4991* (COL) • corregimiento Junín; vereda Gualte; small patch of forest along Hwy Junín - Barbacoas (3–5 KM north of Junín), western slopes of the Cordillera Occidental;1.55987778°N, 78.2198083°W; 910 m; 17 May 2013; fl; *J.L. Clark, L. Clavijo, O. Marín, & H. García 13609* (CAUP, COL, CUVC, HUA, PSO, SEL, US) • vereda Gualte; trail from north side of Hwy Junín-Barbacoas towards Río Ñambí, western slopes of the Cordillera Occidental; 1.368333°N, -78.083056°W; 775 m; 14 May 2013; bud; *J.L. Clark et al. 13500* (COL, SEL) • carretera entre Altaquer y Junin, Cuyamba; 1450 m; 17 Nov 1967; *L.E. Mora O. 4170* (COL) • Ricaurte; La Planada Reserve, 7 km from Chucunes; 1.083333°N, 78.01667°W; 1800 m; 22 Dec 1987; st; *A. Gentry & P. Keating 59691* (MO) • La Planada, trail to El Hondón, 6–12 km SW of La Planada; 1.066667°N, 78.03333°W; 1750–1800 m; 5 Jan 1988; bud; *A. Gentry 60390* (LPB, MO, US) • Trail from La Planada to Pielapi, wet lower montane cloud forest; 1.066667°N, 78.03333°W; 1600–1800 m; 22 July 1988; st; *A. Gentry 63678* (MO) • Reserva Natural La Planada; Ricaurte, trail behind Centro Cientifico leading to mountain top, 1.1666667°N, 77.96667°W; 1830–1930 m, 3 Mar 1989 (bud) *J.F. Smith & M. Galeano 1516* (COL, WIS) • Reserva Natural La Planada; 7 km above Chucunés (along road between Tuquerres and Ricaurte) along trail to El Hondón, beginning at Quebrada Tejón and for 0.5 km beyond, 1.1333333°N, 77.9°W; 780–800 m, 15 Mar 1990, *T. Croat 71474* (PSO) • Reserva Natural La Planada; 7 km de Chucunés; 1.166667°N, -77.96667°W; 1800 m; 13 Dec 1987; bud; *O de Benavides 9024* (MO, US) • ibid.; 27 Sep 1989; fl; *O. de Benavides 10942* (MO, US) • ibid.; Camino a Pialapí; 1.166667°N, 77.96667°W; 1800 m; 21 May 1992 ; fl, fr; *R. Giraldo 138* (HUA) • ibid.; Trocha al Hondón; 1.166667°N, 77.96667°W; 1800 m; Oct 1995 ; fl; *R. Giraldo 12* (HUA) • ibid.; El Hondón; 1.166667°N, 77.96667°W; 12 Apr 1994; *H. Mendoza 595* (COL) • ibid.; 1.1525°N, 77.992833°W; 1800 m, 2 Mar 1995; fl; *H. Mendoza 778* (PSO) • ibid.; 22 Feb 1993, *C.A. Agudelo 2967* (COL) • Reserva Natural La Planada; 1.1525°N, 77.992833°W; 16 Jan 1990; bud; *O. de Benavides 11147* (MO) • ibid.; 1800 m; 1 Feb 1993; *M. Amaya 221* (COL) • ibid.; 1 Sep 1993; *M. Amaya 283* (COL) • Vicinity Ricaurte, along rio Imbí, ca 2–3 km above Ecopetrol Campamento Palmar, located 3 km NW of Ricaurte, along trail to Ramos (indigenous settlement); 1.133333°N, 77.93333°W; 1150 m; 16 Mar 1990; bud; *T. Croat 71528* (PSO) • Camino Las Cruves-Curcuel; 1.133333°N, 77.85°W; 1700–1800 m; 4 Nov 1995; fl; *M.S. González, B.R. Ramírez & A. Muñoz 1274* (PSO) • Resguardo Indígena Pialapí-Pueblo Viejo; Reserva Natural La Planada; sendero Natural El Tejón; 1.1581624°N, 77.981004°W; 1700–1850 m; 19 Jul 2011; fl; *L. Clavijo & C. Caicedo 1600* (COL, CUVC, PSO). **Ecuador. – Carchi.** • Stream by Rafael Quindí’s finca flowing into Río Verde, above Untal (along road to Chical), 0.5 km from finca; 0.8833333°N, 78.133333°W; 1730 m; 25 Nov 1987; fl; *W.S. Hoover & S. Wormley 1532* (MO) • Stream by Rafael Quindí’s finca flowing into Río Verde, above Untal (along road to Chical); 0.8833333°N, 78.133333°W; 1730 m; 25 Nov 1987; fl; *W.S. Hoover & S. Wormley 1548* (MO) • collections from forest area along trail from Rafael Quindí’s house to his mountain finca; 0.8666667°N, 78.133333°W; 1890 m; 28 Nov 1987; *W.S. Hoover & S. Wormley 1899* (MO) • embankments along Río Verde, from point at which trail from Rafael’s mountain finca crosses river, 1.5 km.; 0.8666667°N, 78.133333°W; 1890 m; 29 Nov 1987; fl ; *W.S. Hoover, 1922* (MO) • ibid.; *W.S. Hoover, 1995* (MO) • trail from Paílon to Gualpi Chico area of Awá Reservation, 1.5 km past Río Blanco; 0.85°N, 78.266667°W; 1000–1450 m; 14 Jan 1988; fl; *W.S. Hoover et al. 2434* (MO, US) • trail to Pailon encampment, Gualpi Chico area of Awá Reserve; 0.9666667°N, 78.266667°W; 1350–1400 m; 21 Jan 1988; bud, *W.S. Hoover et al. 3601* (MO) • up small mountain SW of Rafael Quindí’s finca along small stream and descending mountain trail; 0.8666667°N, 78.133333°W; 1930–2100 m; 28 Nov 1987; *W.S. Hoover & S. Wormley* 1813 (MO) • Tulcan, parroquia Chical, Reserva Drácula (Fundación EcoMinga), trail from Chical along Río Blanco and then to summit of Cerro Oscuro; 0.83322°N, 78.2335°W; 2224 m; 17 Mar 2016; fl; *J.L. Clark, S. Ginzbarg & H. Yela 15000* (ECUAMZ, QCA, UNA, US) • Chical; Cerro Golondrinas, ridgeline from campsite to base of Golondrinas; 0.8621861°N, 78.1674667°W; 2200 m; 25 Jan 2024; fl; *J.L. Clark et al. 18123* (QCA, SEL) • ibid.; Cerro Golondrinas, trek from main road (km 22) to campsite; 0.892463889°N, 78.19465833°W; 1650–1900 m; 22 Jan 2024; bud; *J.L. Clark, J. Mia & E. Nolan 17947* (QCA, SEL) • ibid.; collection made along path from the village of Quinyal towards an area known locally as “Gualpi” (near the border of the Reserva Awa); 0.965083333°N, 78.22258333°W; 1200–1700 m; 6 Dec 2001; bud; *J.L. Clark & O. Mejia 6297* (MO, QCNE, SEL, UNA, US). – **Esmeraldas.** • Eloy Alfaro; Reserva Ecológica Cotacachi – Cayapas, parroquia Luis Vargas Torres, Río Santiago, estero Angostura; 0.816666667 S, 78.75°W; 250 m; 8 Dec 1993; bud; *M. Tirado et al. 772* (MO, US) • Quinindé; Bilsa Biological Station, Mache Mountains, 35 km W of Quinindé, 5 km W of Santa Isabel; 0.35°N, 79.73333333°W; 400–600 m; 15 Jul 1996; bud, *J.L. Clark et al.* 2860 (QCNE, SEL, US) •ibid.; Mache-Chindul Ecological Reserve, Bilsa Biological Station, 35 km W of Quinindé; 0.35°N, 79.73333333°W; 500 m; 2 Oct 1996; bud, *J.L. Clark 3007* (QCA, QCNE, US) • San Lorenzo; parroquia Alto Tambo, mature forest 4–8 km west of El Cristal, 0.837778°N, 78.51778°W; 1500–1650 m, 27 May 2008; bud; *J.L. Clark, J, Melton, O. Solarte 10301* (QCNE, UNA, US)• ibid.; parroquia Alto Tambo, mature forest 4–8 km west of El Cristal; 0.837778°N, 78.51778°W; 1500–1650 m, 27 May 2008, *J.L. Clark, J, Melton, O. Solarte 10302* (QCNE, UNA, US) • ibid.; parroquia Alto Tambo, finca Bufalito (Empresa Golden Land), 10–15 km NW of Lita; 0.8747222°N, 78.486944°W; 900–1300 m; 27 Mar 2003; bud; *J.L. Clark & R. Hall 7601* (QCNE, US). – **Imbabura.** • Ibarra; parroquia Lita, comunidad San Francisco, next to Río Verde (13 air-km south of Lita); 0.755833°N, 78.4525°W; 900–1100 m; 24 Mar 2003; fl; *J.L. Clark, R. Hall & F. Nicolalde 7521* (MO, QCNE, SEL, UNA, US) • ibid.; parroquia Lita, unpaved road heading south, near Rocafuerte (between San Gerónimo and La Carolina), accessed via the Lita - San Lorenzo highway (KM 98), road recently developed through the SolGold mining concession (Alpala), finca de Rene Chavez, known locally as “La Esperanza” and adjacent to Río Verde; 0.7330556°N, 78.373889°W; 1550–1700 m; 25 Jul 2022; *J.L. Clark et al. 17143* (MO, NY, SEL, US). – **Pichincha.** • Quito; parroquia Pacto, Mashpi Lodge (Reserva Mashpi), 0.158333°N, 78.8855°W; 1000 m; 16 Mar 2019; *J.L. Clark & L. Jost 16320* (UNA, US) • parroquia Pacto, Mashpi Lodge (Reserva Mashpi); 0.158333°N, 78.8855°W; 788 m, 25 Mar 2024; fl; hiking trail to Río Magnolia; *J.L. Clark, S. Enriquez, S.G. Clark, A. Clark & C. Correa 18587* (QCA, SEL, US).

## Supplementary Material

XML Treatment for Drymonia quadrangulata

## ﻿Additional information

### Author ORCIDs

Laura Clavijo https://orcid.org/0000-0002-3009-9158

John L. Clark https://orcid.org/0000-0002-1414-6380

### Data availability

All of the data that support the findings of this study are available in the main text.
